# Parenchymal and Stromal Cells Contribute to Pro-Inflammatory Myocardial Environment at Early Stages of Diabetes: Protective Role of Resveratrol

**DOI:** 10.3390/nu8110729

**Published:** 2016-11-16

**Authors:** Monia Savi, Leonardo Bocchi, Roberto Sala, Caterina Frati, Costanza Lagrasta, Denise Madeddu, Angela Falco, Serena Pollino, Letizia Bresciani, Michele Miragoli, Massimiliano Zaniboni, Federico Quaini, Daniele Del Rio, Donatella Stilli

**Affiliations:** 1 The Laboratory of Phytochemicals in Physiology, Human Nutrition Unit, Department of Food Science, University of Parma, 43125 Parma, Italy; monia.savi@unipr.it (M.S.); letizia.bresciani@unipr.it (L.B.); 2 Department of Life Sciences, University of Parma, 43124 Parma, Italy; leonardo.bocchi@unipr.it (L.B.); serena.pollino@studenti.unipr.it (S.P.); massimiliano.zaniboni@unipr.it (M.Z.); 3 Department of Biomedical, Biotechnological and Translational Sciences, University of Parma, 43126 Parma, Italy; roberto.sala@unipr.it (R.S.); caterina.frati@unipr.it (C.F.); costanzaannamaria.lagrasta@unipr.it (C.L.); denise.madeddu@unipr.it (D.M.); angela.falco@unipr.it (A.F.); 4 Department of Clinical and Experimental Medicine, University of Parma, 43126 Parma, Italy; federico.quaini@unipr.it; 5 Humanitas Clinical and Research Centre, Rozzano, 20089 Milan, Italy; Michele.Miragoli@humanitasresearch.it; 6 NNEdPro Global Centre for Nutrition and Health, St John’s Innovation Centre, Cowley Road, Cambridge CB4 0WS, UK

**Keywords:** cytokines, diabetes, polyphenols, cardiac cell compartments, cardiomyocyte mechanics, intracellular calcium dynamics

## Abstract

**Background:** Little information is currently available concerning the relative contribution of cardiac parenchymal and stromal cells in the activation of the pro-inflammatory signal cascade, at the initial stages of diabetes. Similarly, the effects of early resveratrol (RSV) treatment on the negative impact of diabetes on the different myocardial cell compartments remain to be defined. **Methods:** In vitro challenge of neonatal cardiomyocytes and fibroblasts to high glucose and in vivo/ex vivo experiments on a rat model of Streptozotocin-induced diabetes were used to specifically address these issues. **Results:** In vitro data indicated that, besides cardiomyocytes, neonatal fibroblasts contribute to generating initial changes in the myocardial environment, in terms of pro-inflammatory cytokine expression. These findings were mostly confirmed at the myocardial tissue level in diabetic rats, after three weeks of hyperglycemia. Specifically, monocyte chemoattractant protein-1 and Fractalkine were up-regulated and initial abnormalities in cardiomyocyte contractility occurred. At later stages of diabetes, a selective enhancement of pro-inflammatory macrophage M1 phenotype and a parallel reduction of anti-inflammatory macrophage M2 phenotype were associated with a marked disorganization of cardiomyocyte ultrastructural properties. RSV treatment inhibited pro-inflammatory cytokine production, leading to a recovery of cardiomyocyte contractile efficiency and a reduced inflammatory cell recruitment. **Conclusion:** Early RSV administration could inhibit the pro-inflammatory diabetic milieu sustained by different cardiac cell types.

## 1. Introduction

The occurrence of ventricular dysfunction independent of coronary artery disease, hypertension and valvular or congenital heart disease has been recognized for over 40 years in both type 1 and type 2 diabetes [1]. Although different mechanisms have been proposed for the development of the diabetic cardiomyopathy (DCM) phenotype, most available data suggest that cellular oxidative stress and moderate inflammation constitute early cardiac responses to hyperglycemia and associated metabolic alterations [2,3,4].

At the initial stages of diabetes, oxidative stress coupled with the activation of pro-inflammatory and cell death pathways, and the inactivation of pro-survival pathways, results in complex biochemical and structural alterations of myocardial tissue, playing a pivotal role in triggering the development of DCM [2,5,6,7,8]. Hence, many efforts have been made to test novel therapeutic strategies aimed at preventing these early detrimental changes in the diabetic heart. In this context, a growing body of evidence supports that administration of low doses of resveratrol (RSV: trans-3,5,4′-trihydroxystilbene), a polyphenolic compound and naturally occurring phytoalexin, may prevent the occurrence of DCM [7,9,10,11,12]. It has been shown that besides its antioxidant, antiapoptotic and anti-inflammatory effects, RSV possesses several other cardio-protective roles due to a large array of direct and indirect target molecules mediating its biological actions. These include modulation of enzyme activity, cell signaling pathways and gene expression [9,10,11,12,13,14,15].

During the past decades, major attention has been devoted to defining diabetes-induced initial changes in functional myocardium, while early alterations in the stromal cell compartment, have been poorly investigated. This is particularly relevant when considering cardiac fibroblasts which constitute the most numerous cell type in the heart, approximately two-thirds of the total cardiac cell number [16], and can affect cardiomyocyte function through both direct and paracrine actions [16,17,18]. It is well known that the functional efficiency of cardiac fibroblasts is severely impaired by chronic diabetes, in terms of proliferation, protein expression, and differentiation into the myofibroblast phenotype, and they largely participate in the increase in collagen and other matrix protein deposition in the diabetic heart [19,20,21]. Conversely, little information is currently available concerning the involvement of cardiac fibroblasts in the activation of the pro-inflammatory signal cascade occurring in the initial phases of diabetes, well before the transition towards overt DCM. Importantly, potential positive effects of early RSV treatment on different myocardial cells and their contribution to the local “diabetic milieu” remain to be defined.

In the present investigation, in vitro and ex vivo approaches were used to specifically address these issues. The in vitro study aims at (i) defining the relative contribution of cardiomyocytes and fibroblasts to the myocardial diabetic “milieu” in terms of pro-inflammatory cytokine expression; and (ii) verifying the ability of RSV active metabolites to reduce the activation of the cellular pro-inflammatory response. In the cell cultures, RSV metabolites were used at the concentration reached at plasma level following in vivo chronic administration of the compound [7]. The cellular model was then validated in diabetic rats, either untreated or subjected to daily intraperitoneal administration of RSV, by analyzing myocardial tissue inflammation and the impact of micro-environmental changes on cardiomyocyte functional properties.

## 2. Materials and Methods

The investigation was approved by the Veterinary Animal Care and Use Committee of the University of Parma-Italy (Prot. No. 59/12) and conforms to the National Ethical Guidelines of the Italian Ministry of Health and the Guide for the Care and Use of Laboratory Animals (National Institute of Health, Bethesda, MD, USA, revised 1996). All experiments were carried out in accordance with the approved guidelines. Before sacrifice all animals were anesthetized with ether (neonatal rats) or ketamine chloride (Imalgene, Merial, Milan, Italy; 40 mg/kg i.p.) plus medetomidine hydrochloride (Domitor, Pfizer Italia S.r.l., Latina, Italy; 0.15 mg/kg i.p.) (adult animals).

### 2.1. In Vitro Study

#### 2.1.1. Isolation and Culture of Neonatal Cardiomyocytes (nCMs) and Cardiac Fibroblasts (nCFs)

Neonatal normal rats (one to two days old) were anesthetized with ether. Following decapitation, the hearts were excised and the ventricles were minced and incubated in a dissociation buffer. The buffer contained Hanks’ Balanced Salt Solution (HBSS, without Ca^2+^ and Mg^2+^; Lonza, Cambridge, UK) supplemented with 1% penicillin/streptomycin (Sigma, Milan, Italy), 0.1% trypsin (Lonza) and 120 µg/mL pancreatin (Sigma). After stirring of the suspension at 35 °C for 15 min, tissue pieces were allowed settling by gravity and the supernatant containing the dissociated cells was transferred into centrifuge tubes which were kept cold on ice. New dissociation buffer was added to the tissue pieces and the entire process was repeated until the tissue was completely digested (three to four cycles). After dissociation, cardiomyocytes were purified by a pre-plating method, which takes advantage of the finding that cardiomyocytes require a longer time to attach to a cell culture dish than other cells in the myocardium, such as fibroblasts. The freshly isolated myocardial cells were plated on uncoated cell-culture dish and cultured for 2 h at 37 °C. The supernatant containing the cardiomyocyte suspension was then collected and filtered into a sterile centrifuge tube (50 µL) whereas fresh medium was added to fibroblasts attached to the bottom of the dish.

Eventually, cardiomyocytes were counted and seeded at a density of 1000 cells/mm^2^ on 35 × 10 mm tissue culture dishes (Greiner Bio-One, Frickenhausen, Germany) treated with collagen (Sigma-Aldrich). Final preparations consisted of (i) homocellular isotropic monolayers of neonatal rat ventricular cardiomyocytes and (ii) isotropic monolayers of fibroblasts (200–400 cells/mm^2^).

The preparations were kept in normoglucidic media (Dulbecco’s Modified Eagle’s Medium, DMEM, Lonza; glucose 5.5 mmol/L) or hyperglucidic media (Iscove’s Modified Dulbecco’s Medium, IMDM, Sigma; glucose 25 mmol/L), with 5% fetal bovine serum (Sigma), 1% penicillin/streptomycin (Lonza), and with or without RSV active metabolites: RSV-glucoronide (1 µmol/L) or RSV-sulphate (1 µmol/L) (Bertin Pharma, Montigny-le-Bretonneux, France) for 21 days. nCM media were changed every 48 h, and nCF media every week. In the last 48 h, the preparations were cultured in the same media at low serum concentration (0.1%). Then, the supernatants harvested from the different cultures were analyzed in order to determine the levels of different pro-inflammatory cytokines and growth factors (RayBio Rat Cytokine Antibody Array I; RayBiotech, Norgross, GA, USA; Table 1).

The concentration of RSV metabolites applied in the set of in vitro experiments was chosen based on our previous works [7,22] in which plasma/urine levels and dose-/time-dependent bioaccumulation of RSV metabolites in the heart tissue were measured, in diabetic rats subjected to daily intraperitoneal injection of trans-RSV (5 mg/kg). We opted for the highest plasma concentration reached by the main identified metabolite, i.e., the sulphate, which peaked at 1 µmol/L [7]. To compare the efficacy of the two main RSV metabolites, we used the same concentration for RSV-glucuronide (1 µmol/L), although the plasma level of this metabolite was much lower (9–45 nmol/L). This approach allowed us to compare the effect of the two previously identified metabolites, knowing that a wide variability has been reported in animals and humans in the framework of phase II hepatic transformations [23].

#### 2.1.2. Molecular Analysis of Conditioned Media

The conditioned medium harvested from each culture was evaluated in order to simultaneously detect the expression level of different cytokines with high specificity [22,24]. Briefly, each membrane was first treated overnight with a blocking buffer at 4 °C and then, after removing the buffer solution, incubated for 2 h with 1 mL of conditioned medium at room temperature (RT). Subsequently, membranes were washed twice and incubated at RT for 2 h with the adjunct of 1 mL of primary biotin-conjugated antibody. Membranes were then incubated with 2 mL of horseradish peroxidase-conjugated streptavidin at RT for 2 h and subsequently developed by using enhanced chemiluminescence-type solution (Merck-Millipore, Darmstadt, Germany) and exposed to Kodak X-Omat AR film. The intensities of signals were quantified after densitometric scanning with ImageQuant software (Molecular Dynamics). For each spot, the net gray level density was determined by background subtracting from the total raw density level. Then, data from the different membranes were normalized to the intensity of the positive control printed onto each membrane (biotin-conjugated IgG). To normalize array data, the membrane exposed to control media was chosen as the reference array. Finally, the measured cytokine level was normalized to the cell number, in each experimental condition. Data were expressed as densitometric units (DU).

### 2.2. In Vivo/Ex Vivo Study

The in vivo/ex vivo study was performed on rats with Streptozotocin-induced diabetes either untreated or subjected to daily RSV administration, in order to (i) validate the in vitro model, by analyzing tissue inflammation and cell damage after three weeks of hyperglycemia; (ii) assess the impact of changes in tissue environment on cardiomyocyte mechanics; (iii) evaluate the effects of RSV treatment in preventing early tissue inflammation by targeting different cell types; and (iv) define the functional counterpart of this cardioprotective action.

#### 2.2.1. Animals, Housing and Treatment

The study population consisted of 63 male Wistar rats (*Rattus norvegicus*) aged 12–14 weeks, weighing 362 ± 5 g, individually housed in a temperature-controlled room at 22–24 °C, with the light on between 7 a.m. and 7 p.m. The bedding of the cages consisted of wood shavings; food and water were freely available. In 46 animals, diabetes was induced by a single intraperitoneal (i.p.) injection of Streptozotocin (STZ, 60 mg/kg; Sigma) while the remaining 17 control rats (group CTR) were injected with saline vehicle (0.9% NaCl). Glucose blood levels and body weights were measured in 4-h-fasting animals, before STZ or vehicle injection, two days after injection, and then weekly until sacrifice. Diabetic animals were either untreated (*n* = 24) or subjected to administration of trans-resveratrol (Sigma), at the concentration of 5 mg/kg/day (*n* = 22; i.p. injection). Only diabetic animals were subjected to RSV administration given that preliminary experiments did not show any effect of three-week RSV treatment on functional parameters in normal animals (Figures S1 and S2). The RSV concentration was selected based on our previous studies in which the dose-time dependent effects of different low doses of the compound on cardiac structure and function have been analyzed, in the same model of diabetes [7,22]. We showed that 5 mg/kg/day constitutes the highest dose capable to induce an almost complete recovery in hemodynamic parameters and cardiomyocyte contractile properties. RSV was dissolved in ethanol to prepare a stock solution (RSV concentration: 12.5 mg/mL) and stored in the dark at 4 °C. For each animal, just before i.p. injection, an appropriate aliquot was taken from the stock solution and diluted in PBS to reach the desired concentration in a final volume of 200 µL. The treatment started immediately after the documented increase in glucose blood levels (two days after STZ injection; blood glucose cut-off: 250 mg/dL).

In selected subgroups of animals (Table 2), cardiomyocyte mechanical properties, cell damage and tissue inflammation were measured three weeks after the induction of hyperglycemia (untreated group: D3; RSV-treated group: D3_RSV), as described below. This time point is generally recognized as a transition phase towards the occurrence of DCM phenotype, characterized by the appearance of first signs of left ventricular (LV) dysfunction and substantial cell loss [7,22].

The remaining animals were evaluated after eight weeks of hyperglycemia (D8 and D8_RSV groups, respectively) (Table 2) to qualitatively investigate, by immunohistochemistry and transmission electron microscopy (TEM), the progression of tissue inflammation, and the potential long-term protective effects of RSV treatment. Indeed, it has been already shown that, at this time, severe LV dysfunction develops resulting in overt DCM phenotype which can be reverted by chronic RSV administration, in the same rat model of diabetes [7,12].

#### 2.2.2. Isolation of Adult Left Ventricular Cardiomyocytes

From the heart of 10 CTR, eight D3, and six D3_RSV rats, individual LV myocytes were enzymatically isolated by collagenase perfusion, as previously described [25].

#### 2.2.3. Cardiomyocyte Contractility and Ca^2+^ Transients

Mechanical properties of freshly isolated ventricular myocytes were assessed by using the IonOptix fluorescence and contractility systems (IonOptix, Milton, MA, USA). LV myocytes were placed in a chamber mounted on the stage of an inverted microscope (Nikon-Eclipse TE2000-U, Nikon Instruments, Florence, Italy) and superfused (1 mL/min at 37 °C) with a Tyrode solution containing (in mmol/L): 140 NaCl, 5.4 KCl, 1 MgCl_2_, 5 HEPES, 5.5 glucose, and 1 CaCl_2_ (pH 7.4, adjusted with NaOH). Only rod-shaped myocytes with clear edges and average sarcomere length ≥1.7 µm were selected for the analysis. All the selected myocytes did not show spontaneous contractions.

The cells were field stimulated at a frequency of 0.5 Hz by constant current pulses (2 ms in duration, and twice diastolic threshold in intensity; MyoPacer Field Stimulator, IonOptix). Load-free contraction of myocytes was measured with the IonOptix system, which captures sarcomere length dynamics via a Fast Fourier Transform algorithm. A total of 250 isolated ventricular myocytes were analyzed (91 from CTR hearts, 93 from D3, 71 from D3_RSV) to compute the following parameters: mean diastolic sarcomere length, fraction of shortening (FS), maximal rates of shortening and re-lengthening (±dL/dt_max_), time to peak of shortening (peak-T), and time at 10% and 90% of re-lengthening (T-rel_10%_, T-rel_90%_). Steady-state contraction of myocytes was achieved before data recording by means of a 10 s conditioning stimulation. Sampling rate was set at 1 kHz.

In a subset of cells of each group (37 from CTR hearts, 48 from D3, 66 from D3_RSV), Ca^2+^ transients were measured simultaneously with cell motion. Ca^2+^ transients were detected by epifluorescence after loading the myocytes with fluo-3-AM (10 µmol/L; Invitrogen, Carlsbad, CA, USA) for 30 min. Excitation length was 480 nm, with emission collected at 535 nm using a 40X oil objective lens (NA: 1.3). Fluo-3 signals were expressed as normalized fluorescence (f/f0: fold increase). The time course of the fluorescence signal decay was described by a single exponential equation, and the time constant (Tau) was used as a measure of the rate of intracellular Ca^2+^ clearing [26].

#### 2.2.4. Analysis of LV Myocardium: Inflammation and DNA Damage in Early Diabetes

Electrophoretic and immunoblot assay (2 CTR, 4 D3, and 4 D3_RSV). The heart was excised, and the left and right ventricles were weighed and immediately frozen at −80 °C. Based on data obtained by in vitro experiments, the expression levels of the following cytokines were determined: MCP-1 (rabbit polyclonal ANTI-MCP1 antibody, 1:1000, Abcam, Cambridge, UK), Fractalkine (rabbit polyclonal ANTI-CX3CL1 antibody, 1:1000, Abcam), LIX (rabbit polyclonal ANTI-LIX1L antibody, 1:1000, Genetes International, Hsinchu City, Taiwan), and VEGF (rabbit polyclonal ANTI-VEGF antibody, 1:1000, Jomar Life Research, Victoria, Australia) as indexes of activation of pro-inflammatory signal cascades in ventricular myocardium.

The left ventricular tissue was mechanically fragmented in liquid nitrogen, and lysed with 300 µL of lysis buffer containing the following (all from Sigma): protease (1:100) and phosphatase (1:100) inhibitors, NaCl (150 mmol/L), Tris-HCl (50 mmol/L), EDTA (5 mmol/L), Nonidet P-40 (1%), sodium fluoride solution (10 mmol/L), sodium diphosphate dibasic (10 mmol/L), SDS (0.1%) and sodium deoxycholate (0.5%). For each animal, equivalents of 50 µg of protein were separated by 10% SDS-PAGE. Membranes were blocked with 5% milk in Tris-Buffered Saline Tween-20 (TBS-T, Sigma) and incubated overnight at 4 °C with the primary antibody. After washing the membranes, a second incubation was performed for one hour at RT with peroxidase conjugated affinity purified goat anti-rabbit secondary antibody (goat Anti-Rabbit IgG Horseradish Peroxidase Conjugate, 1:5000; Bio-Rad, Hercules, CA, USA). Peroxidase activity was developed using the ECL Western blotting system (Amersham, Rahn AG, Zürich, Switzerland), according to the instructions of the manufacturer. Blots were scanned and the intensity of bands was quantified by means of the ImageJ software (NIH, Bethesda, MD, USA). Actin (anti-actin rabbit polyclonal antibody, 1:5000, Sigma) was used as the loading control. More than one gel was used for determining the average value of each protein because our set-up for WB assay allows running only eight samples simultaneously. Thus, in order to obtain measurements in triplicate for every cytokine in each sample (CTR = 2; D3 = 4, D3_RSV = 4), we run the samples together with the internal control several times. In addition, for each run, the 2 CTR samples were always present.

Immunohistochemistry. In each animal, the abdominal aorta was cannulated, the heart was arrested in diastole by injection of CdCl_2_ solution (100 mmol/L, i.v.), and the myocardium was retrogradely perfused with 10% buffered formalin solution. The heart was then excised and placed in formalin solution (10%) for 24 h and embedded in paraffin. Five-µm-thick sections were analyzed under fluorescence microscopy to determine the effects of diabetes and RVS treatment on myocardial cells in terms of DNA lesions. The density of myocardial cells labelled by gamma histone H2AX (γH2AX), a sensitive biomarker of DNA double strand breaks [27], was determined using specific antibodies (rabbit polyclonal anti-γ-H2AX antibody, 1:50; Bethyl Laboratories, Montgomery, TX, USA), in 3 CTR, 4 D3, and 4 D3_RSV hearts. Cardiomyocytes were identified by staining the same sections with monoclonal mouse anti-α-sarcomeric actin antibody (anti-α-SARC; 1:100; Sigma). FITC, TRITC-conjugated anti-mouse or anti-rabbit secondary antibodies (Jackson Laboratory, Baltimore, PA, USA) were used to detect simultaneously the different epitopes. Nuclei were recognized by DAPI (4′,6-diamidine-2-phenyndole; Sigma) staining. Quantitative measurement of γH2AX^pos^ cells was obtained by analyzing a sample area ranging from 8.82 mm^2^ to 16.12 mm^2^ and expressed as the number of positive cells per unit area. For each sample from a minimum of 794 to a maximum of 1694 nuclei were counted. Myocardial cells which were not labelled by α-sarcomeric actin antibody were computed as interstitial cells.

Optimal immunostaining of MCP-1 on control and experimental tissues was obtained by immunoperoxidase. Conversely, for Fractalkine double immunofluorescence was critical to assess its expression in specific myocardial cell types, namely cardiomyocytes and cardiac fibroblasts.

MCP-1: after inhibition of the endogenous peroxidases by dipping the sections in 3% H_2_O_2_ for 10 min, sections were incubated with and rabbit anti-MCP-1 (1:50, Abcam, ab25124) specific primary antibodies. The reaction was revealed using peroxidase conjugated streptavidin that catalyzes the oxide-reduction reaction of the chromogenic substrate (DAB, 3-3′diaminobenzidine; DAKO) resulting in brownish precipitate. All sections were counterstained with Hematoxylin.

Fractalkine: the expression of the cytokine in the LV myocardium was evaluated by immunofluorescence using specific antibody rabbit polyclonal anti-CX3CL1 (1:50, Abcam, ab25088) antibody. To assess the localization of Fractalkine in cardiomyocytes and fibroblasts, anti-α-SARC (Sigma) and monoclonal mouse anti-Vimentin (1:50, Merck Millipore, Vimodrone, Milan, Italy) antibodies were respectively employed. To simultaneously detect multiple antigens, sections were then exposed to FITC, TRITC and Cy5 conjugated secondary antibodies (Jackson Laboratory, Baltimore, PA, USA). Nuclei were recognized by DAPI (Sigma) staining.

Positive controls were represented by immunostaining of sections from the rat kidney for MCP-1 and from bronchial epithelium and rat infarcted myocardium for Fractalkine. Negative controls were represented by omitting primary antibodies from the reaction.

#### 2.2.5. Analysis of LV Myocardium in Late Diabetes: Macrophage Recruitment and Ultrastructural Damage

The analysis at later stages of diabetes was used to confirm previous data on long-term protective effect of the treatment and was limited to verify the progression of tissue inflammation and cardiac structural damage [7].

Inflammatory cell recruitment. LV sections have been used to study macrophage recruitment in the myocardium, at later stages of diabetes, in 2 CTR, 5 D8 and 5 D8_RSV rat hearts. Specifically, pro-inflammatory (M1) and anti-inflammatory (M2) macrophages were analyzed, employing anti-CD40 and anti-CD163 antibodies, respectively [28]. Sections were incubated with the primary antibody, mouse monoclonal anti-CD40 (1:50; Ventana Medical System, Tucson, AZ, USA) and mouse-monoclonal anti-CD163 (prediluted; Ventana Medical System) and revealed through biotin-streptavidin-DAB system (Dako, Milan, Italy). Finally, the sections were counterstained with Mayer’s hematoxylin. The reaction was revealed by immunoperoxidase.

Ultrastructural analysis of the LV myocardium by transmission electron microscopy. Samples of LV myocardium from three D8 and three D8_RSV rats were fixed in Karnovsky solution (4% formaldehyde, 5% glutaraldehyde) for 6 h at RT. After washing several times with phosphate buffer (0.1 mol/L, pH 7.2), the tissue were post-fixed in 1% osmium tetroxide (OsO_4_) for 90 min at RT and dehydrated by increasing concentration of alcohol. Then, samples were washed with propylene oxide and embedded in epoxy resin. Sections of 0.5 μm thickness were stained with methylene blue and safranin in order to morphologically select the field of interest. Subsequently, ultrathin sections of 60–80 nm thickness were collected on a 300 mesh copper grid and, after staining with uranyl acetate and lead citrate, were qualitatively examined under a transmission electron microscope (Philips EM 208S; Fei Electron Optics BV, Eindhoven, The Netherlands).

### 2.3. Statistical Analysis

All in vitro experiments were repeated at least three times. Normal distribution of variables was checked by means of the Kolmogorov-Smirnov test. Data are reported as mean ± standard error (SEM). Comparisons among groups involved one-way ANOVA followed by post-hoc individual comparisons with the Bonferroni or Games-Howell test, when appropriate (cell mechanics and immunohistochemistry), and non-parametric statistical test Kruskal Wallis and U-Mann Whitney test (western blot data) (IBM-SPSS 22.0, SPSS Inc., Chicago, IL, USA). One-way ANOVA with Holm-Šídák post test was used to compare cytokine levels detected in the culture media (GraphPad Software, San Diego, CA, USA; www.graphpad.com). Student’s *t*-test was used for comparing the density of macrophage cell types, in D8 and D8_RSV groups. Differences were considered statistically significant at *p* < 0.05.

## 3. Results

### 3.1. In Vitro Study

Conditioned media were harvested from the different cell cultures, i.e., nCFs or nCMs maintained in normoglucidic or hyperglucidic conditions, with or without RSV metabolites, for three weeks. Only cytokines whose levels consistently showed a significant increase of at least two-fold in a hyperglycemic environment compared to a normoglycemic were considered significant and included in the results.

**Figure 1:**
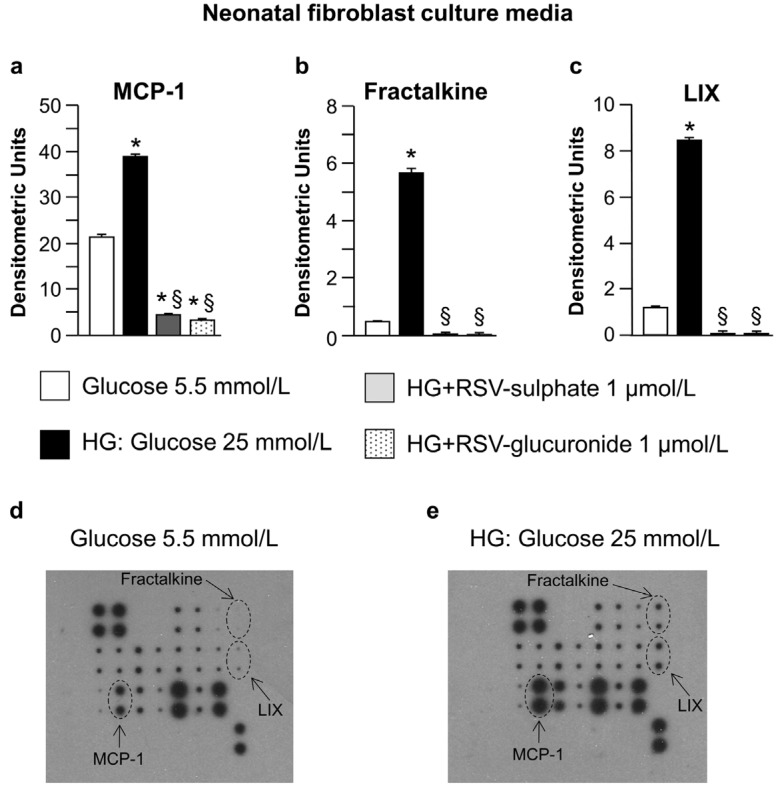

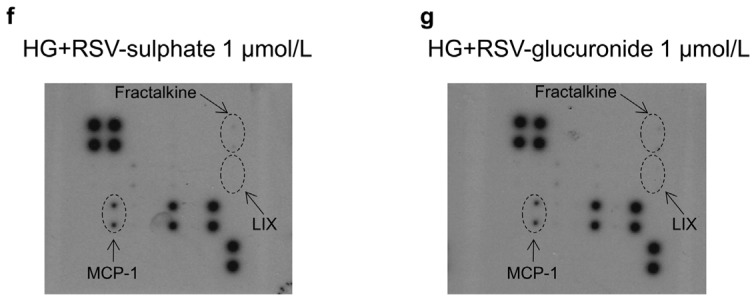
Analysis of neonatal fibroblast culture media. Average values ± standard error of the mean SEM of pro-inflammatory cytokines expressed by neonatal cardiac fibroblasts (**a**–**c**) cultured in normoglucidic (Glucose 5.5 mmol/L), hyperglucidic (HG, High Glucose: 25 mmol/L) or hyperglucidic + RSV metabolites medium. * *p* < 0.05 vs. normoglucidic conditions. § *p* < 0.05 vs. HG conditions (one-way ANOVA, Holm-Šídák post test); Panels (**d**–**g**) typical images obtained with RayBio Rat Cytokine Antibody Array 1. The membranes were probed with conditioned media from neonatal cardiac fibroblasts cultured in different conditions. Membranes were exposed to Kodak X-Omat AR film. MCP-1: Monocyte chemotactic protein-1; LIX: Lipopolysaccharide-inducible CXC chemokine.

**Figure 2:**
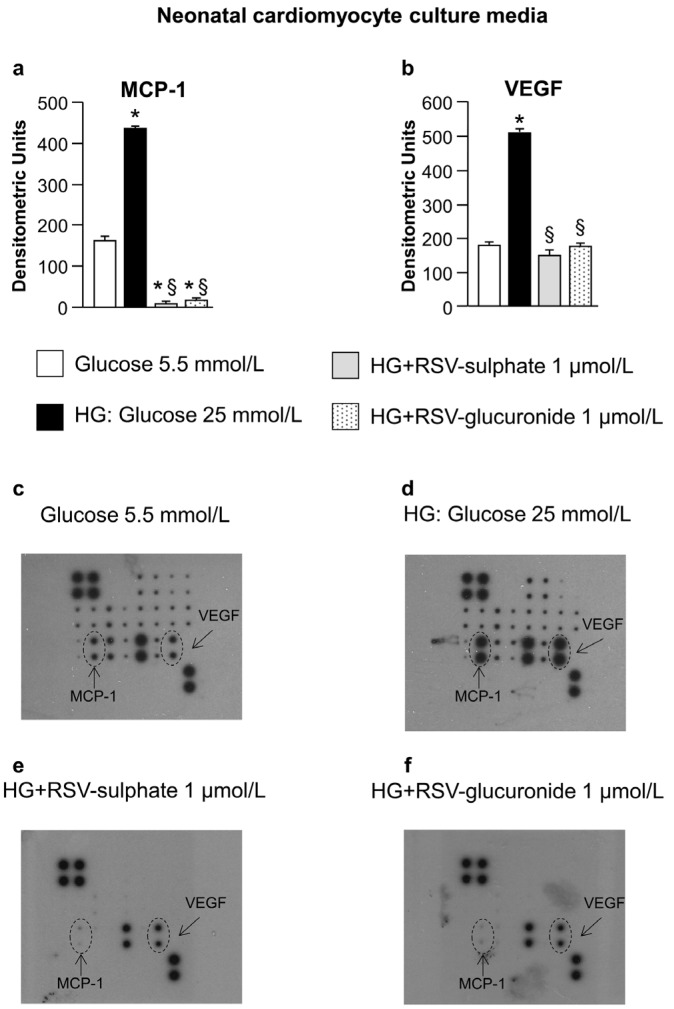
Analysis of neonatal cardiomyocyte culture media. Average values ± SEM of pro-inflammatory cytokines expressed by neonatal cardiomyocytes (**a**,**b**) cultured in normoglucidic (Glucose 5.5 mmol/L), hyperglucidic (HG, High Glucose: 25 mmol/L) or hyperglucidic + RSV metabolites medium. * *p* < 0.05 vs. normoglucidic conditions. § *p* < 0.05 vs. HG conditions (one-way ANOVA, Holm-Šídák post test); Panels (**c**–**f**) typical images obtained with RayBio Rat Cytokine Antibody Array 1. VEGF: Vascular endothelial growth factor. Other explanations as in Figure 1.

Both nCFs and nCMs released high levels of the pro-inflammatory cytokine monocyte chemoattractant protein-1 (MCP-1) when cultured in hyperglucidic in comparison with normoglucidic medium (Figure 1a and Figure 2a). nCFs also produced high levels of Fractalkine and lipopolysaccharide-induced CXC chemokine (LIX) (Figure 1b,c) while the VEGF concentration increased only in nCM culture media (Figure 2b). The changes in cytokines induced by high glucose were significantly reduced by adding RSV metabolites (Figure 1a–c and Figure 2a,b). It is noteworthy that the adjunct of RSV metabolites decreased MCP-1 levels even in comparison with normoglucidic conditions, in both nCF and nCM cultures (Figure 1a and Figure 2a), suggesting that the compounds can also counteract the moderate cell oxidative stress due to the “unnatural environment” of culture systems.

### 3.2. In Vivo/Ex Vivo Study

#### 3.2.1. Effects of Diabetes and RSV Treatment on LV Myocardial Tissue: Inflammation and DNA Damage

**Figure 3:**
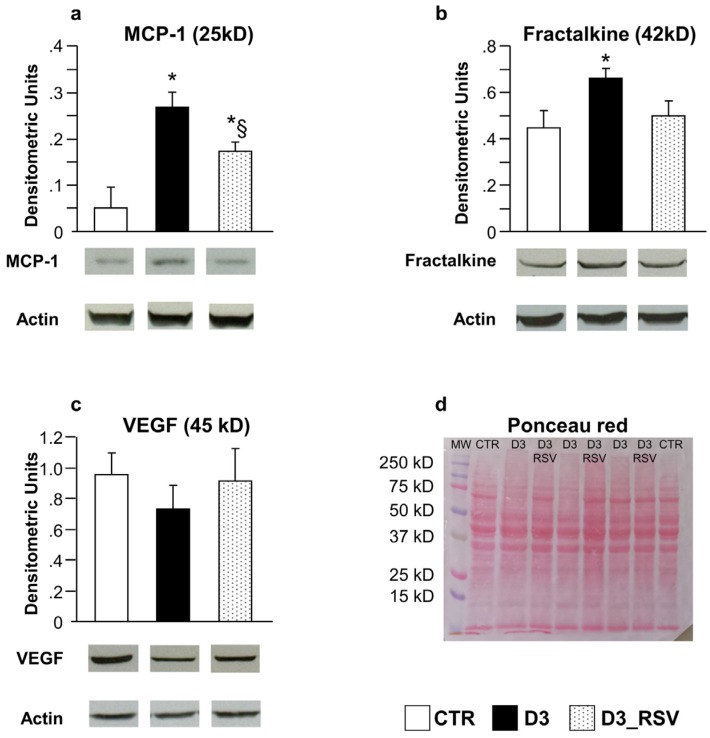
RSV-treatment reduced diabetes-induced tissue inflammation. Mean values ± SEM of MCP-1 (**a**), Fractalkine (**b**) and VEGF (**c**) expression levels in LV myocardium of control (CTR), diabetic (D3) and RSV-treated diabetic rats (D3_RSV), after three weeks of hyperglycemia (Western blot assay). Quantitative comparisons were performed on data derived from different gels processed in parallel (see Methods). Sets of bands related to cytokine expression of three animals representative of the average behavior observed in each group are reported at the bottom of the corresponding graph. Lanes were not adjacent in the gel, as indicated by the white space between them. The visualization of all proteins in one membrane stained with Ponceau Red is reported in (**d**), together with the molecular weights. * *p* < 0.05 vs. CTR, § *p* < 0.05 significant differences between D3 and D3_RSV (Kruskal-Wallis analysis of variance and Mann Whitney U-test).

In vivo/ex vivo experiments have been performed to determine whether the effects of high glucose and RSV metabolites adjunct on cultured neonatal cells can be translated to the in vivo setting. Specifically, to validate the in vitro data, the expression levels of MCP-1, Fractalkine, LIX and VEGF were analyzed on myocardial ventricular tissue from control animals and RSV-treated or untreated diabetic rats, after three weeks of hyperglycemia. The results confirmed that MCP-1, the best-known chemotactic cytokine implicated in the development of cardiomyopathy [29,30,31], was essentially undetectable in control hearts while it was significantly increased in the LV myocardium of untreated D3 rats and consistently decreased in RSV-treated animals (D3_RSV group; Figure 3a). Similar results were obtained when Fractalkine expression was measured (Figure 3b). It is noteworthy that Fractalkine, besides its action as a chemoattractant, possesses a direct detrimental effect on cardiac cells, independently of inflammatory cell recruitment [32,33]. In contrast, VEGF signals exhibited comparable values in all experimental groups (Figure 3c), with only minor changes. LIX cytokine could not be detected in control and diabetic hearts, independently of the treatment.

**Figure 4:**
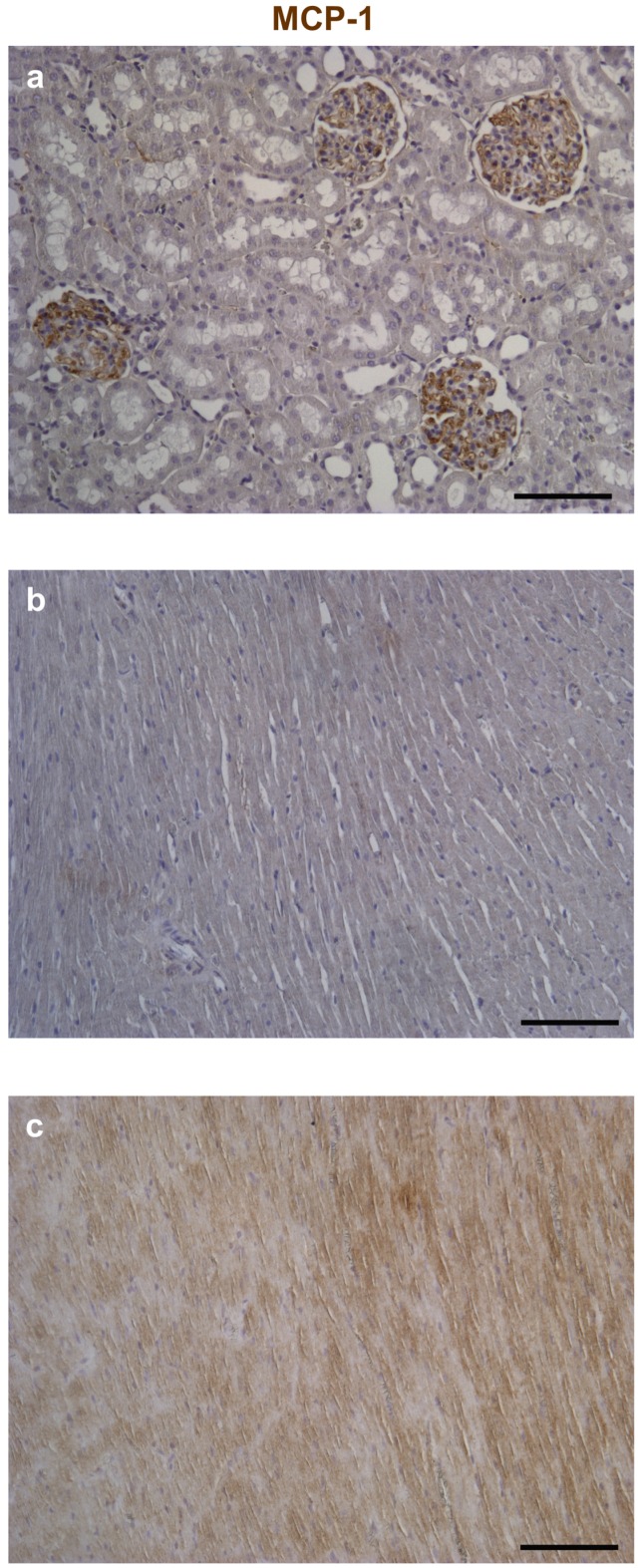
Immunohistochemical analysis of Monocyte Chemoattractant Protein-1 (MCP-1). Reference tissues for the detection of MCP-1 by immunoperoxidase (brownish) were represented by the rat kidney (**a**); Compared to control (**b**); Higher expression of the chemokine is apparent in both cardiomyocytes and interstitial cells of the diabetic left ventricular (LV) myocardium (**c**). Nuclei were counterstained by Hematoxylin. Scale bars: 100 µm.

**Figure 5:**
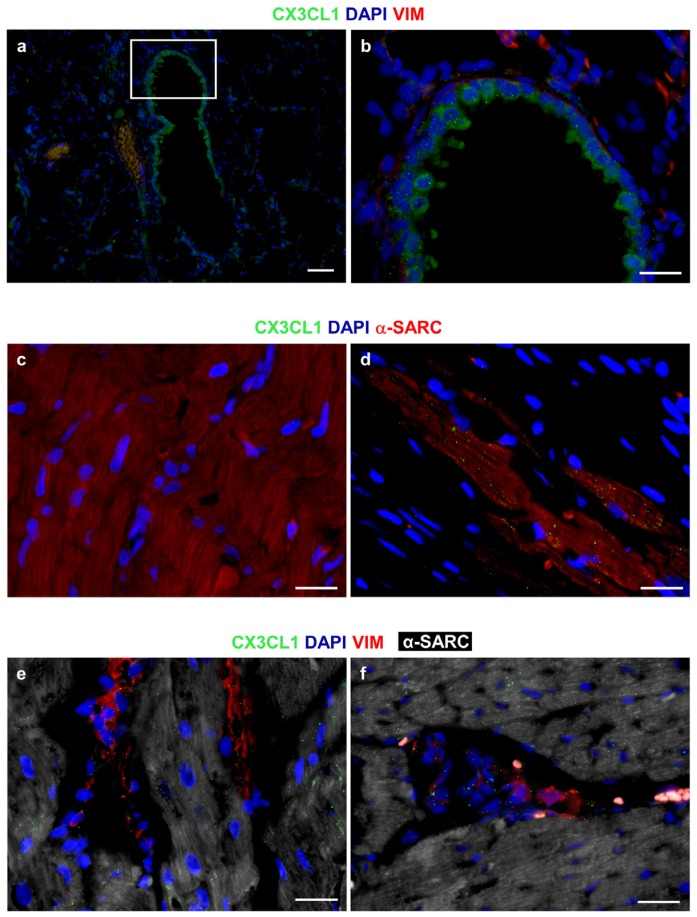
Immunofluorescence analysis of CX3CL1 (Fractalkine). The granular and diffuse cytoplasmic expression of CX3CL1 (green) is apparent in bronchiolar epithelial cells of the rat lung, used as a positive control tissue; (**a**,**b**) The white square includes an area shown at higher magnification in b where interstitial cells are depicted by the red fluorescence of vimentin (VIM); (**c**) Internal negative control. Absence of immunofluorescence signals in a section of the rat diabetic heart exposed to FITC secondary antibodies in the absence of primary anti-CX3CL1 antibodies. Cardiomyocytes are recognized by the red fluorescence of alpha-sarcomeric actin (α-SARC); (**d**) A rather dense granular, dot-like pattern of CX3CL1 (green) expression in α-SARC (red) positive cardiomyocytes is illustrated in a section of the rat infarcted myocardium; (**e**,**f**) A granular, dot-like expression of Fractalkine (green) is apparent in VIM (red) positive fibroblasts and interstitial cells surrounding α-SARC (withe) positive cardiomyocytes. The pro-inflammatory cytokine is also detectable in cardiomyocytes. (**a**–**f**): Blue fluorescence corresponds to DAPI staining of nuclei. Scale Bars: (**a**) = 50 µm, (**b**–**f**) = 20 µm.

Fractalkine and MCP-1 were also detected by immunohistochemistry in myocardial sections and respective positive control tissues (Figure 4 and Figure 5). Tissue analysis showed a higher expression of both cytokines in cardiomyocytes and stromal compartments of the diabetic myocardium compared to CTR (Figure 4 and Figure 5). It is noteworthy that Fractalkine could be specifically detected in cardiac fibroblasts and cardiomyocytes, thus confirming the early contribution of the two cell types to the microenvironmental inflammatory bulk induced by three weeks of hyperglycemia.

**Figure 6:**
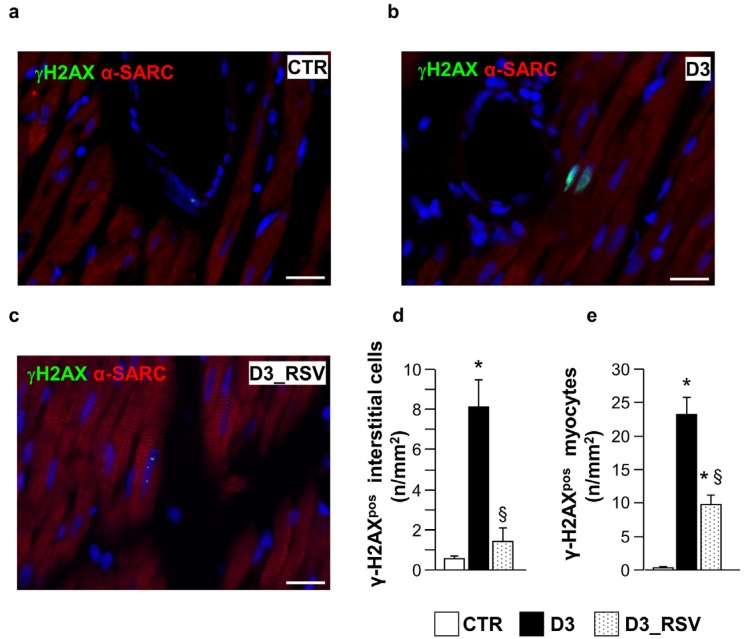
RSV treatment reduced diabetes-induced DNA damage. Panels a–c: detection of DNA double strand breaks in sections of the LV myocardium from control (**a**), untreated (**b**) and RSV-treated (**c**) diabetic rats, by immunofluorescence. Nuclear labeling by antibodies against gamma-Histone2 AX (green, γH2AX) is documented in cardiomyocytes recognized by the red fluorescence of α-sarcomeric actin (red). Two cardiomyocytes show diffuse nuclear fluorescence in the D3 heart while dot-like signals are present in a cardiomyocyte from RSV-treated myocardium. Blue fluorescence corresponds to DAPI staining of nuclei. Scale bars: 20 µm; In (**d**–**e**), bar graphs illustrating the density of γH2AX positive interstitial cells (**d**) and cardiomyocytes (**e**), in control (CTR), untreated (D3) and RSV-treated (D3_RSV) diabetic myocardium. Data are reported as mean ± SEM * *p* < 0.05 vs. CTR; § *p* < 0.05 significant differences between D3 and D3_RSV (one-way ANOVA, Games-Howell post-hoc test).

The pro-inflammatory tissue environment of diabetic rats was associated with DNA damage in the LV myocardium of untreated diabetic animals, as indicated by the higher density of nuclear γH2AX labeling in cardiomyocytes and interstitial cells in comparison with CTR (Figure 6). The DNA double-strand break is considered one of the earliest events involved in the DNA damage response to different stimuli, including oxidative stress, accelerating cell senescence. In turn, general features of senescent cells involve changes in morphology, chromatin organization and gene expression, and the secretion of many inflammatory modulators [27]. RSV administration significantly reduced the density of γH2AX labeling in both cellular compartments (Figure 6b–d).

#### 3.2.2. RSV Lowers the Detrimental Effects of Diabetic Environment on Cell Mechanics and Ca^2+^ Transients

The extensive analysis of mechanical properties in diabetic cardiomyocytes revealed that the abnormalities observed in the myocardial environment have a functional counterpart.

**Figure 7:**
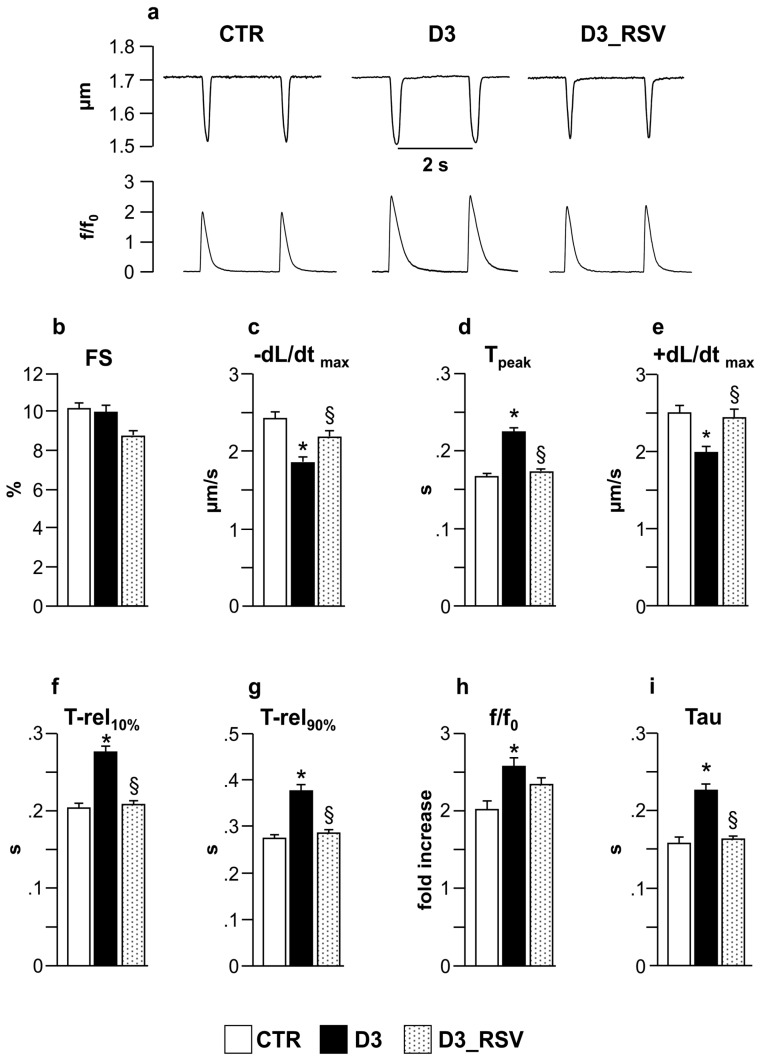
RSV treatment significantly restored cell mechanics and Ca^2+^ handling. (**a**) Representative examples of sarcomere shortening and corresponding Ca^2+^ transients (normalized traces: fold increase) recorded from CTR, D3 and D3_RSV ventricular myocytes, after three weeks of hyperglycemia; In bar graphs (**b**–**i**): mean values ± SEM of cardiomyocyte mechanical properties. FS: fraction of shortening (**b**); −dL/dt_max_: maximal rate of shortening (**c**); T_peak_: duration of cell contraction (**d**); +dL/dt_max_: maximal rate of relengthening (**e**); T-rel_10%_: time to 10% of relengthening (**f**); T-rel_90%_: time to 90% of relengthening (**g**); f/f0: Ca^2+^ transient amplitude expressed as peak fluorescence normalized to baseline fluorescence (**h**); and Tau: time constant of the intracellular Ca^2+^ decay (**i**). * *p* < 0.05: significant differences vs. CTR; § *p* < 0.05: significant differences between D3 and D3_RSV (one-way ANOVA, Games-Howell post-hoc test).

The average diastolic sarcomere length was similar in all groups, approximately 1.7 µm (average value: 1.712 ± 0.002 µm). Conversely, intracellular Ca^2+^ dynamics and contraction/relaxation properties measured in unloaded ventricular myocytes isolated from D3 hearts were markedly impaired compared to CTR (Figure 7). Specifically, D3 cardiomyocytes exhibited a significant decrease in the maximal rate of shortening (−dL/dt_max_, Figure 7c) and relengthening (+dL/dt_max_, Figure 7e), leading to a global prolongation of contraction and relengthening times (T_peak_, T-rel_10%_, T-rel_90%_; Figure 7d,f,g). The compromised contractility was accompanied by a significant increase in the relative (to diastolic values) amount of Ca^2+^ released by the Sarcoplasmic Reticulum SR (f/f0, +30% on average; Figure 7h), and in the time required to remove it from cytoplasm (Tau, +50%; Figure 7i). RSV treatment led to an almost complete recovery of all functional parameters, which attained values comparable to those measured in the CTR group (Figure 7c–i).

#### 3.2.3. Inflammatory Cell Recruitment Occurs at Later Stages of Diabetes

**Figure 8:**
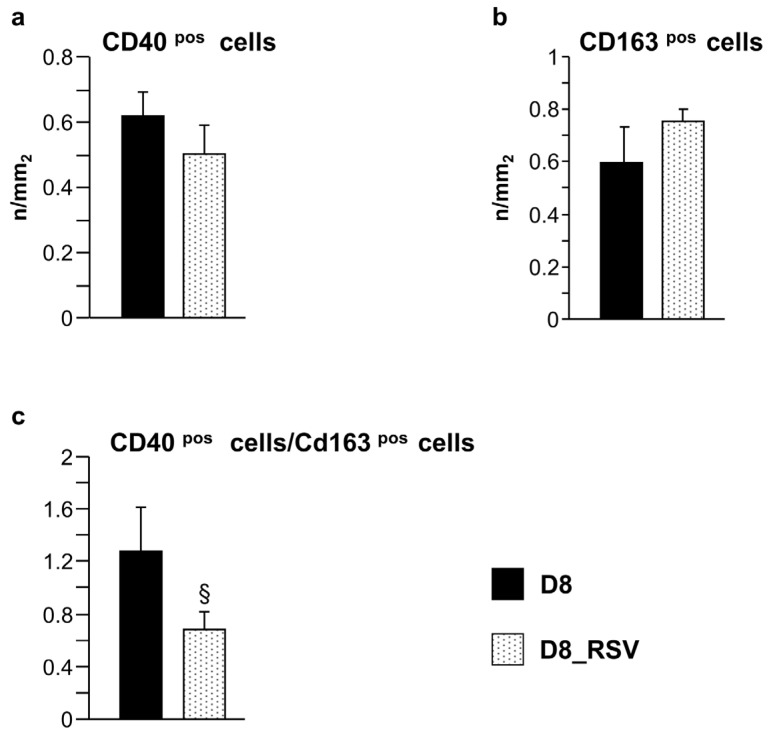
Positive effects of RSV treatment on macrophage recruitment. Mean values ± SEM of CD40pos and CD163^pos^ cell density (**a**,**b**), and CD40^pos^/CD163^pos^ cell ratio (**c**), in the LV myocardium of D8 and D8_RSV rats (immunohistochemistry). § *p* < 0.05, significant differences between D8 and D8_RSV groups (Student’s *t*-test).

In accordance with our previous findings [7], the infiltration of inflammatory cells in diabetic hearts was negligible after three weeks of hyperglycemia, while a substantial increase in macrophage recruitment was found during the progression of the disease (eight weeks of hyperglycemia, D8 group). Macrophages are plastic cells able to switch from a classical pro-inflammatory M1 to an alternative anti-inflammatory M2 phenotype, depending on the environmental stimuli [28]. Using CD40 and CD163 as markers suggestive of the M1 and M2 phenotypes, respectively, both types of macrophages were documented in the LV tissue of untreated D8 animals, while these cells were essentially absent in control hearts. RSV-treated hearts (D8_RSV group) showed a trend towards an enhancement of anti-inflammatory CD163^pos^ macrophages with a parallel partial reduction of pro-inflammatory CD40^pos^ cells (Figure 8a,b), leading to a significant reduction (approximately −50%) in the CD40^pos^/CD163^pos^ macrophage ratio observed in D8 hearts (Figure 8c).

#### 3.2.4. Ultrastructural Alterations of the Myocardium at Later Stages of Diabetes

**Figure 9:**
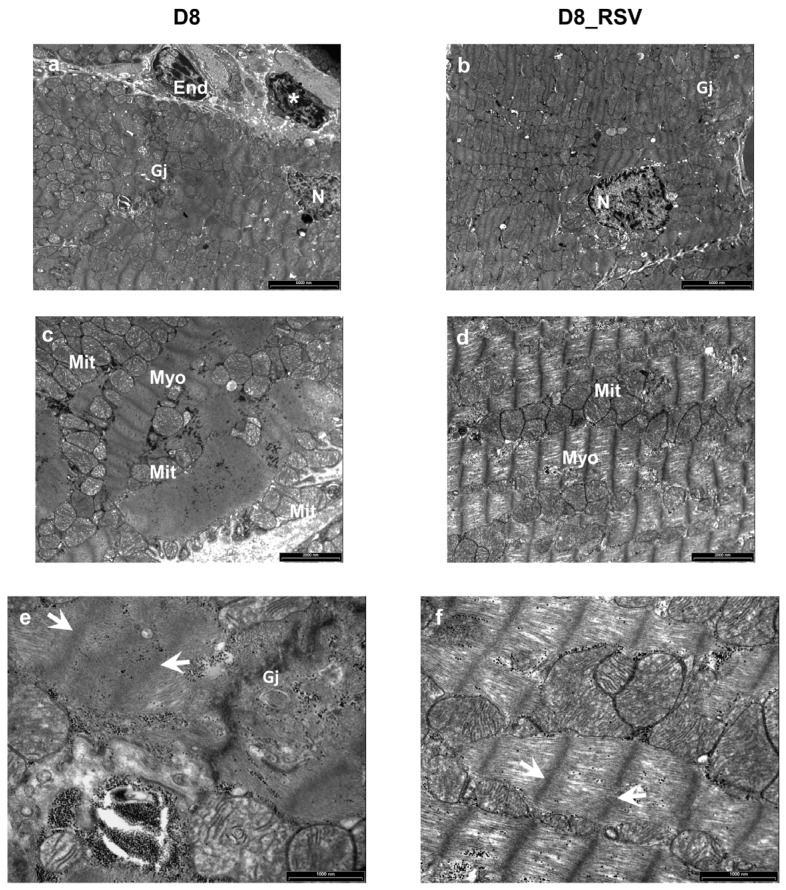
Positive effects of RSV treatment on ultrastructural damage of diabetic LV myocardium. Ultra-thin longitudinal sections of the LV myocardium from untreated (D8) and RSV-treated (D8_RSV) diabetic rats. (**a**,**b**) Low magnification images illustrating cardiomyocytes connected by gap junctions (Gj). An inflammatory cell (*) showing electron-dense granules near a capillary endothelial cell (End) is also documented in the diabetic rat myocardium (**a**). N: cardiomyocyte nuclei. (**c**,**d**) Disorganization and severe alteration of contractile myofibrils (Myo) are illustrated in the diabetic myocardium (**c**) whereas a more preserved sarcomere striation is present in RSV-treated heart (**d**). Mitochondria (Mit) are running parallel to myofibrils in RSV-treated rat myocardium (**d**) which are swollen and clustering between disarrayed contractile filaments in diabetic rats (**c**). At higher magnification, a marked ultrastructural effacement of sarcomeres (white arrows) at the junctional interface (Gj) of diabetic cardiomyocytes (**e**) compared to RSV-treated myocardium (**f**) is apparent. Scale bars: (**a**,**b**): 5 µm; (**c**,**d**): 2 µm; (**e**,**f**): 1 µm.

Ultrastructural analysis by transmission electron microscopy revealed focal alterations of the cardiomyocytes’ morphology in untreated D8 hearts. Partial disorganization of intercellular junctions and substantial sarcomere Z-line and myofibrillar disarray were observed. In addition, impairment of the myofilaments’ integrity (Figure 9a,c,e) associated with moderate mitochondrial damage was present. These ultrastructural alterations were nearly undetectable in the myocardium of RSV-treated diabetic animals after eight weeks of hyperglycemia (Figure 9b,d,f).

## 4. Discussion

Our study suggests that at the initial stages of diabetes (i) interstitial cells in addition to cardiomyocytes promote early unfavorable changes in the myocardial diabetic milieu, in terms of pro-inflammatory cytokine production; (ii) alterations in the myocardial tissue environment are associated with initial DNA damage and the occurrence of abnormalities in cardiomyocyte contractile properties, in the absence of LV structural damage or collagen accumulation; (iii) RSV treatment is able to significantly reduce cytokine expression in myocardial cells, at early stages of the disease, resulting in an almost complete recovery of functional parameters, as documented by both in vitro and in vivo/ex vivo studies.

The infiltration of inflammatory cells occurs at later stages, during the progression of the disease, with a selective enhancement of the pro-inflammatory macrophage M1 phenotype and a parallel reduction of the anti-inflammatory macrophage M2 phenotype. These later events are associated with marked alterations of cardiomyocyte ultrastructural properties. Chronic RSV treatment counteracts the progression of tissue inflammation and prevents structural remodeling. These results indicate that RSV holds great potential to treat diabetes and would be useful to support conventional therapy.

### 4.1. In Vitro Study

The in vitro model was specifically used to evaluate the relative contribution of nCFs and nCMs in promoting changes in the diabetic environment, in terms of pro-inflammatory cytokine expression, and to test the ability of RSV metabolites in attenuating cytokine expression in both cell types. Primary neonatal cardiac cells constitute a biologically relevant model widely used to explore the molecular mechanisms underlying heart diseases and the effects of pharmacological treatments [34], mainly when prolonged culture times are required, as in the present study. The time of exposure to high glucose concentration (three weeks), with or without the adjunct of RSV metabolites, mimicking a dietary RSV supplementation, was based on our previous data on the same model of diabetes [7]. Specifically, we have previously shown in the same animal model that initial signs of impaired cardiac function, as measured at both organ (hemodynamics) and cardiomyocyte (cell mechanics) levels, mainly attributable to cell oxidative stress and moderate tissue inflammation associated with a high degree of apoptotic cell death, take place after three weeks of hyperglycemia, in the absence of collagen accumulation and fibrotic damage.

The analysis of conditioned media indicated that both nCFs and nCMs may contribute to triggering changes in hyperglucidic medium, although some differences between the two cell types have been observed. High glucose induced MCP-1 production by nCFs as well as nCMs, while only nCFs produced high levels of Fractalkine. Higher levels of lipopolysaccharide-induced CXC chemokine (LIX) were also expressed by nCFs cultured in high glucose as compared with normoglucidic conditions, while VEGF levels increased only in the hyperglucidic culture media of nCMs. By taking into consideration that experiments performed on cultured neonatal cardiac cells may not replicate the in vivo environment, we attempted to confirm these findings by the analysis of myocardial tissue samples isolated from diabetic rats.

### 4.2. In Vivo/Ex Vivo Study

In accordance with in vitro data, after three weeks of hyperglycemia, higher levels of MCP-1 and Fractalkine proteins in the LV myocardium were measured compared to controls. Immunohistochemical analysis also revealed that in the adult tissue, the two cytokines were expressed by different cardiac cell types, including myocytes and stromal cells. MCP-1 is known to participate in both recruitment and activation of monocytes and macrophages at sites of inflammation, thus constituting a critical player in the progression of diabetic cardiomyopathy. Infiltrated macrophages can in turn potentiate the inflammatory response by producing cytokines and toxic mediators such as free radicals [35]. Soluble Fractalkine acts as a potent chemo-attractant contributing to the pathogenesis of various inflammatory diseases [32,36]. In addition, it should be outlined that besides the pro-inflammatory action of this cytokine, the binding of Fractalkine on its receptor on cardiomyocytes results in a decreased speed of contraction and relaxation under basal conditions as well as under beta-adrenergic stimulation [33]. It is noteworthy that Fractalkine has been shown to induce up-regulation of different phosphatases, including protein phosphatase 1 (PP1) and protein phosphatase 2A (PP2A), in failing myocardium [37]. PP1 and PP2A dephosphorylate phospholamban, which is a key regulator of SERCA activity and thereby the uptake of Ca^2+^ into the sarcoplasmic reticulum. Thus, it is conceivable that increased Fractalkine expression in the myocardium, resulting in dephosphorylation of phospholamban and reduction in SERCA activity, contributes to a reduction in myocardial contractile function. In accordance with these findings, we found that the altered tissue environment was associated with a significant impairment of cardiomyocyte mechanical properties and intracellular Ca^2+^ dynamics. Our findings are in accordance with previous observations in the same rat model of diabetes [7,12], indicating that abnormal intracellular Ca^2+^ homeostasis can be considered one of the major and early pathological changes in diabetic cardiomyopathy, due to functional alterations in multiple proteins involved in Ca^2+^ release and uptake. The rate of Ca^2+^ transient recovery is commonly measured as a mono-exponential decay [26] which assumes that Ca^2+^ removal, at any time, depends on the cytoplasmic Ca^2+^ concentration, and the time constant of its exponential decay, in the absence of changes in removal mechanisms, is constant, independently of the amplitude of the transient. Thus, the approximately 50% increase in Tau recorded in D3 cells (Figure 7i) does not follow as a simple consequence of the transient amplitude increase, but shows instead a dramatic decreased efficiency of at least one of the Ca^2+^ removal mechanisms. In this regard, a major contribution is expected to be played by Sarcoplasmic Reticulum Ca^2+^ ATP-ase (SERCA), which is predominant in rat ventricular myocytes [12,26]. Consistent with data on cardiomyocyte contractile properties, changes in LV function were observed at the organ level in D3 animals, as indicated by in vivo measured hemodynamic parameters (Figure S3), in accordance with previous studies [7,12,22].

Unlike in vitro results, LIX was absent in the LV tissue of normal and diabetic animals while the VEGF expression exhibited was comparable among groups, with only a slight decline in D3 myocardium as compared with CTR. LIX was shown to play a critical role in neutrophil recruitment and activation of the inflammatory molecular cascade during ischemia-reperfusion injury [38,39,40], in the presence of marked acute oxidative stress. It is conceivable that the degree of oxidative stress developed over three weeks of hyperglycemia is much lower than that following ischemia/reperfusion and is thus unable to activate the expression of LIX by adult cardiac fibroblasts in our model of diabetes. On the other hand, the discrepancy with in vitro observations documenting a high expression of LIX in hyperglucidic medium of fibroblasts could be attributed to a higher sensitivity of nCFs to moderate increases in oxidative stress induced by high glucose.

Concerning VEGF expression in diabetic myocardium, the inconsistency of the data reported in the literature is most likely due to the duration of the disease and the species employed. Sasso et al. [41] demonstrated an increased VEGF expression associated with a down-regulation of its receptors and signal transduction, partially responsible for the reduced neoangiogenesis observed in type-2 diabetic human subjects. Conversely, the majority of data in rodent models of diabetes documented that hyperglycemia is associated with a reduced VEGF isoform expression in the myocardium, potentially contributing to microvascular rarefaction which characterizes DCM in the absence of atherosclerosis [42,43]. Accordingly, in our in vivo model of early diabetes, we found a slight decrease in VEGF expression in D3 hearts, although the difference did not reach statistical significance. The increased VEGF levels measured in hyperglucidic media of cultured nCMs suggests that the expression pattern of VEGF could vary significantly between neonatal and adult cardiomyocytes exposed to hyperglucidic environment. On the other hand, it has been shown in vitro that high glucose induces VEGF expression in several cell types such as retinal pigmented [44], vascular smooth muscle [45], and mesangial [46] cells. In addition, VEGF concentration has been reported to be significantly increased in the developing hearts of the embryos from diabetic mice [47].

### 4.3. Effects of Resveratrol Treatment

The adjunct of RSV metabolites to the culture medium drastically reduced the levels of pro-inflammatory cytokines and VEGF which became virtually undetectable in both nCF and nCM culture media. Although the physiological mechanisms by which RSV exerts its anti-inflammatory action are still a subject of debate, two main intracellular pathways have been proposed as key mediators, including the activation of the NAD-dependent deacetylase SIRT1 and/or AMP-activated protein kinase (AMPK) [10,11,48,49,50]. AMPK and SIRT1 have similar functions in metabolism and cell survival, ultimately leading to the inhibition of nuclear factor kappa B (NF-kB), a key component of the intracellular inflammatory response.

In accordance with in vitro findings, the RSV treatment succeeded in significantly reducing the chemokine concentration in diabetic LV myocardium. The RSV-induced decline in tissue levels of Fractalkine, which plays a key role in worsening cell mechanics [32,33,37], and the activation of SIRT1-dependent transcriptional regulatory mechanisms, which restore SERCA expression and intracellular Ca^2+^ dynamics [12], might explain the almost complete recovery of the cardiomyocyte mechanical properties and hemodynamic performance in RSV-treated diabetic hearts.

The pro-inflammatory cytokines produced by cardiac cells from the early stages of diabetes can be responsible for the enhanced monocyte recruitment in diabetic myocardium observed at later stages of diabetes (D8 group), associated with clear-cut abnormalities of cardiomyocyte ultrastructural properties. In line with these findings, relevant signs of ventricular dysfunction and the overt DCM phenotype occur in this model of late diabetes, as reported in previous studies [12,22]. Although we cannot exclude that other mechanisms, besides humoral changes, are involved [50], our data confirm that prolonged hyperglycemia induces M1 macrophage infiltration which is known to be associated with reduced survival and greater tissue damage [28,51]. RSV treatment promoted the M2 macrophage phenotype, resulting in a significant reduction in the M1/M2 macrophage ratio, accompanied by a recovery of cardiomyocyte ultrastructural properties.

Overall, we showed that RSV attenuates early pro-inflammatory cytokine expression in different cardiac cell types, including cardiomyocytes and fibroblasts. These beneficial effects, combined with the large array of biological actions of the compound, ultimately result in significant cardioprotection preventing the morphofunctional ventricular remodeling of diabetic hearts.

### 4.4. Translational Impact

It should also be noted that the equivalent in humans of the RSV dose used in the present study (daily administration of 5 mg/kg) would correspond approximately to an overall daily intake of 85 mg. Although reaching an intake of 85 mg of RSV through dietary sources is almost impossible, food supplements usually contain relatively high amounts of the compound, ranging from 55 mg to more than 500 mg per capsule [52], making the 85 mg target relatively easy, even considering the bioavailability factor.

## 5. Conclusions

Our results suggest that high glucose per se triggers both cardiomyocytes and interstitial cells, among which fibroblasts constitute the most numerous cell type, to synthesize and release pro-inflammatory chemokines at the early stages of hyperglycemia, thereby providing a mechanism for enhanced cell recruitment and amplification of the inflammatory response. We showed here for the first time that in early diabetes, Fractalkine contributes to the unfavorable myocardial environment and may play a key role in triggering initial changes in cardiomyocyte contractile properties. Further, we demonstrated that RSV administration could inhibit pro-inflammatory cytokine production from different cardiac cell types, resulting in a recovery of contractile efficiency and long-lasting protection of the diabetic heart.

Taken together, these findings suggest a new promising adjuvant therapeutic strategy, which goes beyond the mere ‘cardiomyocyte-centered’ approach for DCM prevention and treatment. In order to translate our experimental observations, additional studies are needed to unveil the specific intracellular pathways responsible for the negative impact of the diabetic milieu on cardiomyocyte contractile machinery and the counteractive action of RSV.

## Figures and Tables

**Table 1:** Cytokine and growth factor antibody array.

	A	B	C	D	E	F	G	H
1	POS	POS	NEG	NEG	CINC-2	CINC-3	CNTF	Fractalkine
2	POS	POS	NEG	NEG	CINC-2	CINC-3	CNTF	Fractalkine
3	GM-CSF	IFN gamma	IL-1 alpha	IL-1 beta	IL-4	IL-6	IL-10	LIX
4	GM-CSF	IFN gamma	IL-1 alpha	IL-1 beta	IL-4	IL-6	IL-10	LIX
5	Leptin	MCP-1	MIP-3 alpha	beta NGF	TIMP-1	TNF alpha	VEGF	BLANK
6	Leptin	MCP-1	MIP-3 alpha	beta NGF	TIMP-1	TNF alpha	VEGF	BLANK
7	BLANK	BLANK	BLANK	BLANK	BLANK	BLANK	BLANK	POS
8	BLANK	BLANK	BLANK	BLANK	BLANK	BLANK	BLANK	POS

Cytokine-Induced Neutrophil Chemoattractant-2 (CINC-2); Cytokine-Induced Neutrophil Chemoattractant-3 (CINC-3); Ciliary neurotrophic factor (CNTF); Granulocyte-macrophage colony-stimulating factor (GM-CSF); Interleukins (IL-1α, IL-1β IL-4, IL-6, IL-10); Interferon gamma (IFN gamma); Lipopolysaccharide-inducible CXC chemokine (LIX); Monocyte chemotactic protein-1 (MCP-1); Macrophage Inflammatory Protein-3 (MIP-3 alpha); Nerve growth factor-beta (β-NGF); Tissue inhibitor of metalloproteinases (TIMP-1); Tumor necrosis factor alpha (TNF alpha); Vascular endothelial growth factor (VEGF). Positive and negative control spots (POS and NEG, respectively).

**Table 2:** In vivo/ex vivo study: Experimental protocol and sampling.

Total Number of Rats: 63	Saline Injection CONTROL (CTR): 17	STZ Injection DIABETIC (D): 46	
Three weeks after injection	CTR: 15	UNTREATED	RSV TREATED
		D3: 16	D3_RSV: 14
Experimental procedures			
Cell mechanics	CTR: 10	D3: 8	D3_RSV: 6
Immunohistochemistry	CTR: 3	D3: 4	D3_RSV: 4
Western Blot	CTR: 2	D3: 4	D3_RSV: 4
Eight weeks after injection	CTR: 2	D8: 8	D8_RSV: 8
Experimental procedures			
Immunohistochemistry (M1-M2)	CTR: 2	D8: 5	D8_RSV: 5
TEM	-	D8: 3	D8_RSV: 3

D3 and D3-RSV: untreated and resveratrol (RSV) treated group, after 3 weeks of hyperglycemia; D8 and D8-RSV: untreated and RSV- treated group, after 8 weeks of hyperglycemia.

## Supplementary Materials

The following are available online at http://www.mdpi.com/2072-6643/8/11/729/s1, Figure S1: Hemodynamic parameters in untreated and RSV-treated normal animals, Figure S2: Contractile properties of cardiomyocytes isolated from untreated and RSV-treated normal animals, Figure S3: RSV treatment significantly restored cardiac hemodynamics in diabetic rats.
