# Supplementary material for: Parenchymal and Stromal Cells Contribute to Pro-Inflammatory Myocardial Environment at Early Stages of Diabetes: Protective Role of Resveratrol
Source: Nutrients. 2016 Nov 16;8(11):729. doi: 10.3390/nu8110729 (PMC5133113; doi:10.3390/nu8110729)
Supplement: Supplementary file 1 [file nutrients-08-00729-s001.docx]

Supplementary Materials: Parenchymal and Stromal Cells Contribute to Pro-Inflammatory Myocardial Environment at Early Stages of Diabetes: Protective Role of Resveratrol

Monia Savi, Leonardo Bocchi, Roberto Sala, Caterina Frati, Costanza Lagrasta, Denise Madeddu, Angela Falco, Serena Pollino, Letizia Bresciani, Michele Miragoli, Massimiliano Zaniboni, Federico Quaini, Daniele Del Rio and Donatella Stilli


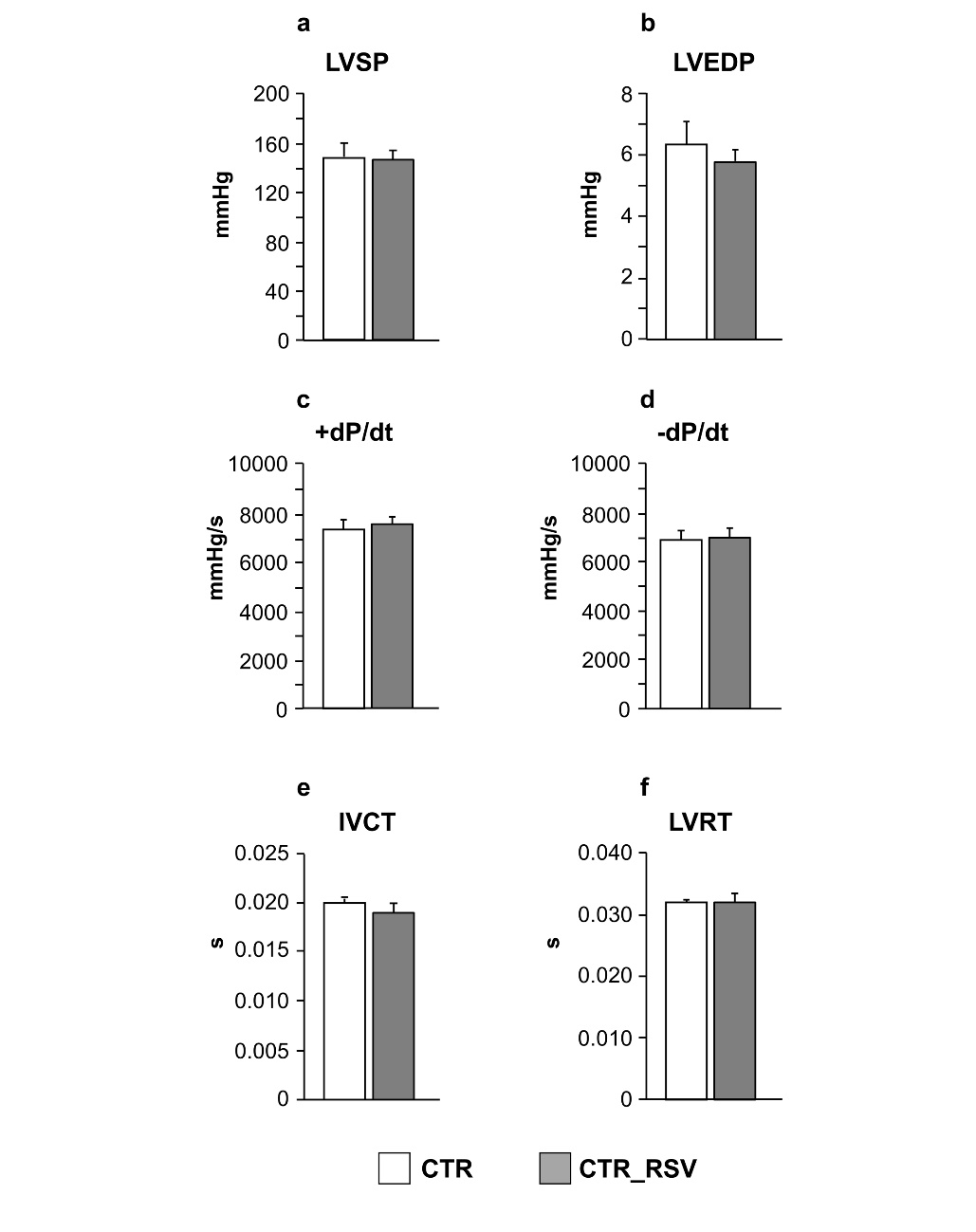


**Figure S1.** Hemodynamic parameters in untreated and RSV-treated normal animals. Mean values ± SEM of hemodynamic parameters recorded in control animals either untreated (CTR) or submitted to RSV administration (CTR_RSV) for 3 weeks (5 mg/Kg/die, i.p.): (**a**) left ventricular systolic pressure (LVSP); (**b**) left ventricular end-diastolic pressure (LVEDP); (**c**) maximum rate of ventricular pressure rise (+dP/dt); (**d**) maximum rate of ventricular pressure reduction (−dP/dt); (**e**) isovolumic contraction time (IVCT); and (**f**) left ventricular relaxation time (LVRT) computed from −dP/dt to 5 mmHg above LVEDP, taken as index of isovolumic relaxation time. No statistically significant difference was observed between the two groups (Student *t*-test).


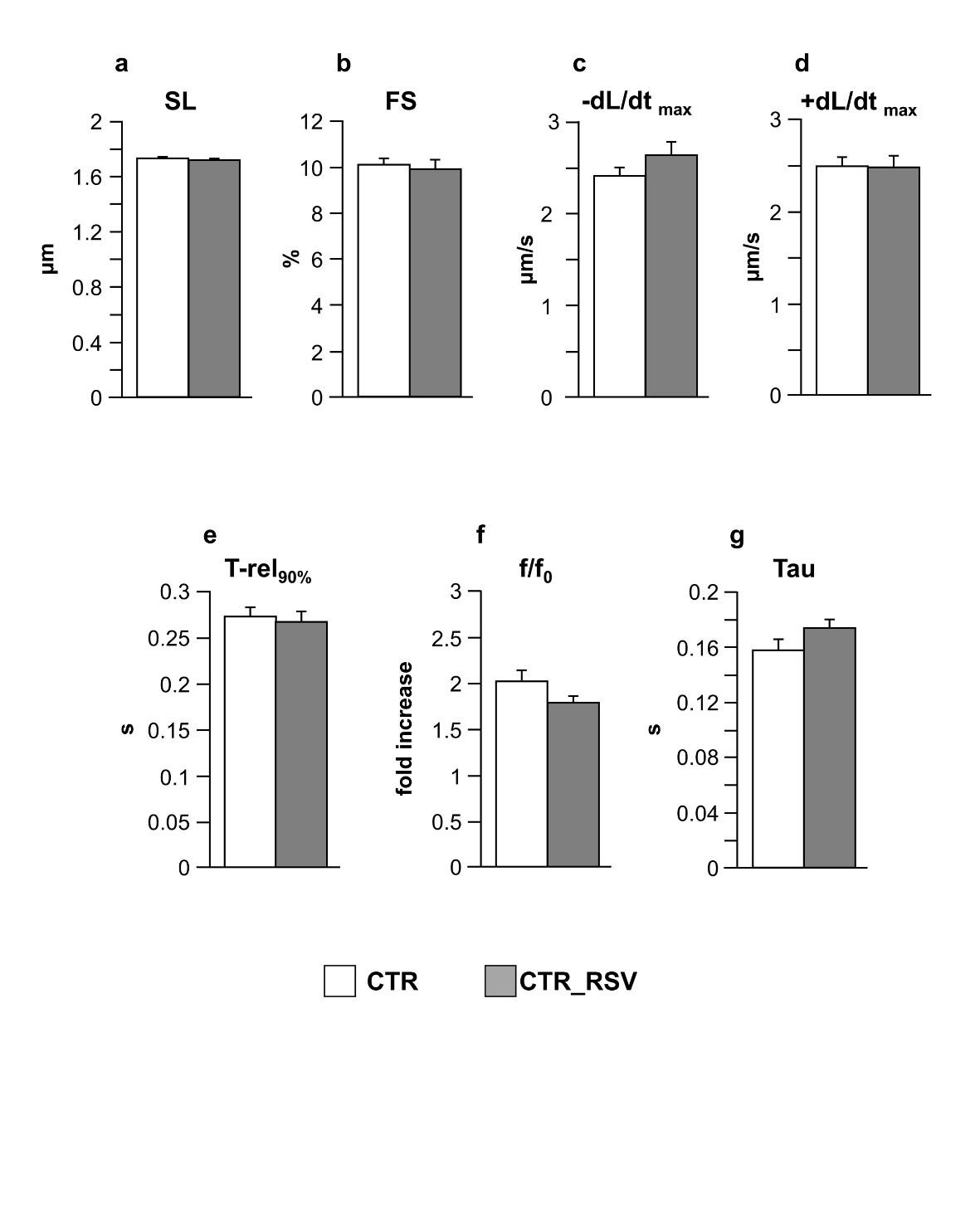


**Figure S2.** Contractile properties of cardiomyocytes isolated from untreated and RSV-treated normal animals. Mean values ± SEM of contractile properties and Calcium transients recorded in cardiomyocytes enzymatically isolated from the heart of untreated (CTR) and RSV-treated (CTR_RSV) normal animals, after 3 weeks of RSV administration (5 mg/Kg/die, i.p.). (**a**) sarcomere length (SL); (**b**) fraction of shortening (FS); (**c**) maximal rate of shortening (−dL/dt_max_); (**d**) maximal rate of relengthening (+dL/dt_max_); _(_**e**) time to 90% of relengthening (T-rel_90%_); (**f**) calcium transient amplitude expressed as peak fluorescence normalized to baseline fluorescence (f/f0); and (**g**) time constant of the intracellular Calcium decay (Tau). No statistically significant difference was observed between the two cell groups (Student *t*-test).


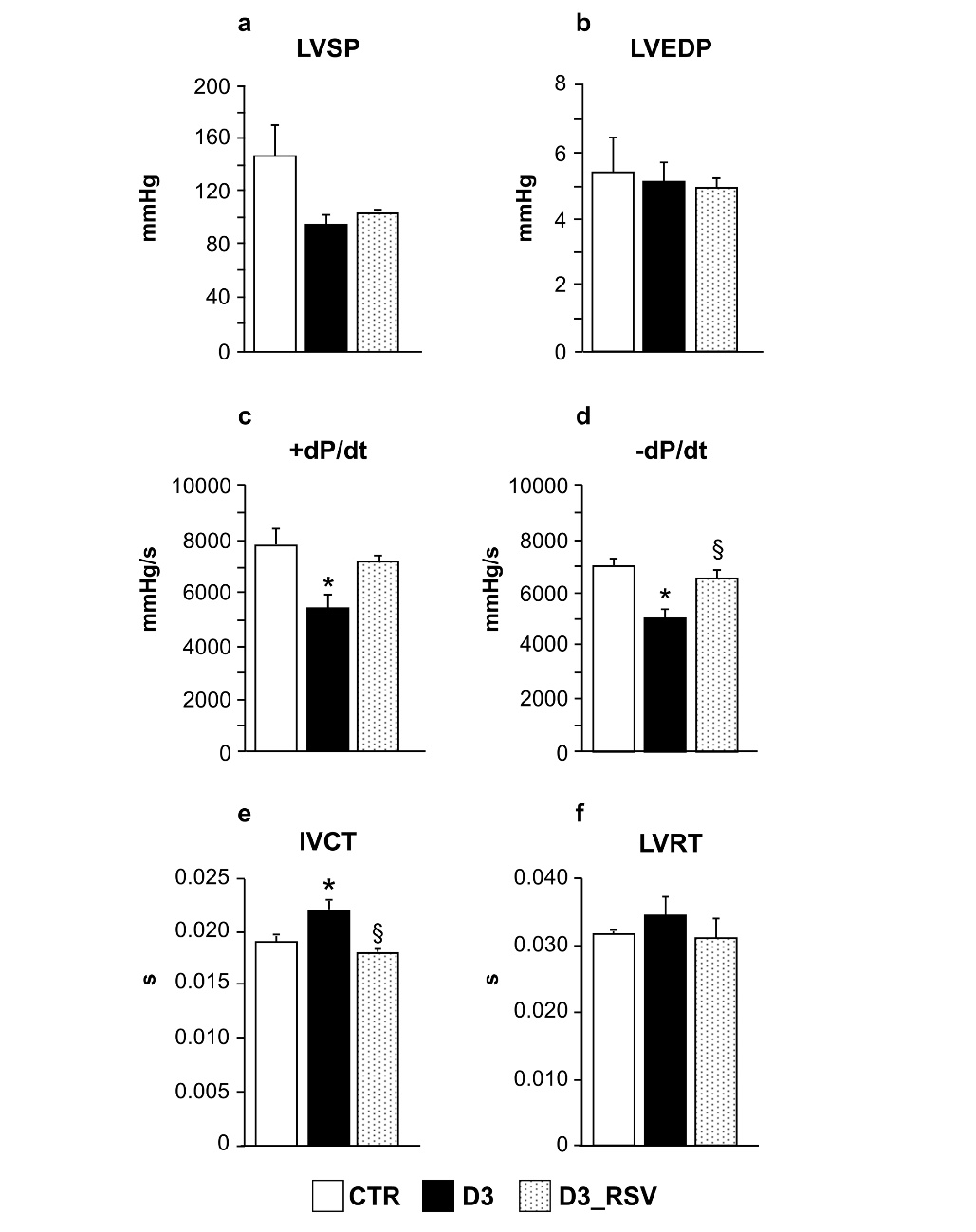


**Figure S3.** RSV-treatment significantly restored cardiac hemodynamics. Mean values ± SEM of hemodynamic parameters recorded in control (CTR), untreated diabetic animals (D3) and RSV-treated diabetic rats (D3_RSV), before cardiomyocyte isolation: (**a**) left ventricular systolic pressure (LVSP); (**b**) left ventricular end-diastolic pressure (LVEDP); (**c**) maximum rate of ventricular pressure rise (+dP/dt); (**d**) maximum rate of ventricular pressure reduction (−dP/dt); (**e**) isovolumic contraction time (IVCT); and (**f**) left ventricular relaxation time (LVRT). * *p* < 0.05 vs. CTR, ^§^ *p* < 0.05 vs. D3.
